# Transcriptome Sequencing and Characterization of Japanese Scallop *Patinopecten yessoensis* from Different Shell Color Lines

**DOI:** 10.1371/journal.pone.0116406

**Published:** 2015-02-13

**Authors:** Jun Ding, Le Zhao, Yaqing Chang, Wenming Zhao, Zhenlin Du, Zhenlin Hao

**Affiliations:** 1 Key Laboratory of Mariculture & Stock Enhancement in North China’s Sea, Ministry of Agriculture, Dalian Ocean University, Dalian, China; 2 Beijing Institute of Genomics, Chinese Academy of Sciences, Beijing, China; University of North Carolina at Charlotte, UNITED STATES

## Abstract

Shell color is an important trait that is used in breeding the Japanese scallop *Patinopecten yessoensis*, the most economically important scallop species in China. We constructed four transcriptome libraries from different shell color lines of *P. yessoensis*: the left and right shell mantles of ordinary strains of *P. yessoensis* and the left shell mantles of the ‘Ivory’ and ‘Maple’ strains. These four libraries were paired-end sequenced using the Illumina HiSeq 2000 platform and contained 54,802,692 sequences, 40,798,962 sequences, 74,019,262 sequences, and 44,466,166 sequences, respectively. A total of 214,087,082 expressed sequence tags were assembled into 73,522 unigenes with an average size of 1,163 bp. When the data were compared against the public Nr and Swiss-Prot databases using BlastX, nearly 30.55% (22,458) of the unigenes were significantly matched to known unique proteins. Gene Ontology annotation and pathway mapping analysis using the Kyoto Encyclopedia of Genes and Genomes categorized unigenes according to their diverse biological functions and processes and identified candidate genes that were potentially involved in growth, pigmentation, metal transcription, and immunity. Expression profile analysis was performed on all four libraries and many differentially expressed genes were identified. In addition, 5,772 simple sequence repeats were obtained from the *P. yessoensis* transcriptomes, and 464,197, 395,646, and 310,649 single nucleotide polymorphisms were revealed in the ordinary strains, the ‘Ivory’ strain, and the ‘Maple’ strain, respectively. These results provide valuable information for future genomic studies on *P. yessoensis* and improve our understanding of the molecular mechanisms involved in the growth, immunity, shell coloring, and shell biomineralization of this species. These resources also may be used in a variety of applications, such as trait mapping, marker-assisted breeding, studies of population genetics and genomics, and work on functional genomics.

## Introduction

The Japanese scallop *Patinopecten yessoensis* is a bivalve that is naturally distributed along the coastline of northern Japan, the northern Korean Peninsula, and the Far East region of Russia. Because of its great commercial value, *P*. *yessoensis* has become one of the most important marine aquacultured shellfish in the north region of China since it was introduced in 1982 [1]. The annual production of scallops reached 1,200,000 tons and 2,000,000 hectares by 2007, accounting for 58 billion yuan of output value in China. More than 50% of this value (200,000 tons and 30 billion yuan of output value) came from *P*. *yessoensis* [2].

Consumers evaluate the quality and value of seafood according to its color, and certain colors often represent high quality. In *P*. *yessoensis*, the colors of the left and right shells are typically reddish-brown and white, respectively, and the left shell is typically smaller than the right shell. A small percentage of *P*. *yessoensis* individuals possess two white shells and display better growth traits than wild-type individuals, despite being raised in similar culture conditions[3]. This fact suggests that the color patterns of scallop shells could be a useful marker for selective breeding. Therefore, our research group’s long-term selective breeding program focused on developing an improved ‘Ivory’ strain of *P*. *yessoensis* in which both shells were completely white, growth rates were rapid, and scallops were tolerant to high temperatures. As an additional point of comparison, our group created another strain, ‘Maple,’ a hybrid of ordinary strain and ‘Ivory’ individuals. This strain has a red and white pattern on the left shell.

The mantle is a unique organ tissue in bivalve mollusks, located midway between the visceral mass and shell. The mantle is in direct contact with the environment and has a sensory function. It can adjust the valves in response to unfavorable environmental conditions[4], and it controls inflow of water into the shell’s internal chamber, which is responsible for respiratory and filter feeding purposes. Another main function of the mantle is to secrete biomineralization proteins to form the shell[5].The mantle pallial and mantle edge are the main secretory tissues.Previous studies on the different shell colors of *P*. *yessoensis* mainly focused on the scallops’ physiological properties [6], genetic structure, and microsatellite markers [7–8].However, the underlying molecular differences and functional consequences of those differences with respect to shell color and performance among strains are unknown, due to the lack of genomic resources available for *P*. *yessoensis*. As expressed sequenced tags (ESTs) provide comprehensive information with respect to the dynamics of the scallops’ transcriptome, it present a valuable resource for breeding and research.

Due to cost and time limitations, it is infeasible to consider whole genome sequencing for this species. Fortunately, RNA-Seq based on next-generation sequencing (NGS) is an option. This is a high-throughput technology that offers great advantages for the examination of the fine structure of a transcriptome [9]. Especially when no genome sequence is available, transcriptome sequencing provides an effective way to obtain large amounts of sequence data [10]. In addition, transcriptome sequences exclude non-coding DNA, so the sequences that are obtained contain a high percentage of functional information, helping to reveal the molecular mechanism of functional genes [11–13]. Moreover, the availability of a large number of genetic markers developed using NGS technologies is facilitating trait mapping and marker-assisted breeding [14].

In this study, we sampled the mantles from different shell color lines of *P*. *yessoensis* and used Illumina paired-end sequencing technology to generate a large EST dataset. Many simple sequence repeats (SSRs) and single-nucleotide polymorphisms (SNPs) were identified. This study is the first characterization of *P*. *yessoensis* from different shell color lines by analyzing large-scale transcriptome sequences. These sequences will serve as a valuable resource for the development of molecular markers, as well as research on gene mapping, comparative genomics, and functional gene discovery.

## Results and Discussion

### Paired-end sequencing and assembly

Four *P*. *yessoensis* cDNA libraries representing different shell color lines were constructed and used for Illumina paired-end sequencing to generate representative transcripts of a wide range of biological processes. The P1 library represents the left shell mantle of the ordinary strain of *P*. *yessoensis*, the P2 library represents the right shell mantle of the ordinary strain, the P3 library represents the left shell mantle of the ‘Ivory’ strain, and the P4 library represents the left shell mantle of the ‘Maple’ strain. A total of 214,105,082 raw reads with an average length of 101 bp were acquired with four libraries. The raw reads produced in P1, P2, P3, and P4 have been submitted to the NCBI SRA database (accession numbers: SRR1185949, SRR1185962, SRR1185963, and SRR1185966). After the low-quality reads were filtered out of the sequence data, 166,521,376 (77.78%) high-quality reads remained and were used for the *de novo* assembly. An overview of the sequencing procedure is presented in S1 Table. Because no reference genome exists for *P*. *yessoensis*, the high-quality reads from all four libraries were combined and assembled into a reference transcriptome using Trinity software[15]. This assembly yielded a total of 73,522 unigenes with an average length of 1163 bp, a minimum length of 300 bp, and a maximum length of 33,371 bp. An overview of the sequencing and assembly process is presented in Table 1.

**Table 1:** Summary statistics of *Patinopectin yessoensis* mantle transcriptome assembly using Trinity software.

Unigene	Max. Length (bp)	Min. Length (bp)	Mean Length (bp)	Median Length (bp)	N50	N90	Total Length (bp)
**Number**	33,371	300	1,163	714	1,768	484	85,514,539

### Functional annotation of the *P*. *yessoensis* transcriptome

Several complementary methods were used to annotate the *P*. *yessoensis* transcriptome assembly. First, the unigenes were compared against the public Nr and Swiss-Prot databases using BlastX (E-value<1e^-5^). A total of 22,458 (30.55%) unigenes were assigned to biological functions, leaving more than half of the unigenes (69.45%) not matched to known genes. The inability to annotate a large percentage of unigenes is likely a consequence of the paucity of sequences available in public databases from phylogenetically closely related species. Similar situations exist in the cases of other marine animals, including *Arctica islandica*(32.84% annotation rate after transcriptome analysis)[16]and *Laternula elliptica*(16.93% annotation rate after transcriptome analysis)[17].

Moreover, the length of query sequences always influences the results of Blast comparison, so short reads obtained from sequencing are seldom matched to known genes [18]. In our study, sequences shorter than 300 bp were eliminated from assembly results in order to ensure that gene matches were meaningful. Among sequences shorter than 999 bp, only 14.54% were annotated, and among sequences longer than 1000 bp, the rate increased to 58.19% (Table 2). Annotation efficiency increased with the length of the sequence.

**Table 2:** Summary statistics of functional annotation of *Patinopectin yessoensis* transcriptome.

Category	All sequences	300–999bp	≥ 1000 bp
**Total number of unigenes**	73,522	46,670	26,852
**Unigene matches against Nr and Swiss-Prot**	22,410	6,787	15,623
**Unigene matches against GO**	13,575	3,226	10,349
**Unigene matches against KEGG**	15,179	3,762	11,417

As for the species distribution of these successfully annotated unigenes, 62.14% of the hits matched to the Bivalvia class in general, including 13,577 (60.45%) sequences that were annotated to proteins from *Crassostrea gigas*; 137 (0.61%) matched to *Azumapecten farreri*; 69 (0.31%) matched to *Mytilus galloprovincialis*; 45 (0.20%) matched to *Argopecten irradians*; 42 (0.18%) matched to *Pinctada fucata*; and 66 (0.30%) matched to another Bivalvia species. In previous work, Hou et al. [19] matched only 4.10% of sequences from the *P*. *yessoensis* transcriptome to the Bivalvia class. This result suggests that as large-scale sequencing of marine animals continues to expand, annotation efficiency will increase. A total of 599 (2.67%) annotated unigenes in this study matched to prokaryotes and protozoans, apparently due to contamination from seawater, and these sequences have been removed. Therefore, a total of 21,859 annotated unigenes (S2 Table) were applied to the subsequent analysis, including the mapping of functional genes, analysis of differential expression, and SSR and SNP mining.

### EuKaryotic Orthologous Groups (KOG) classification

**Fig 1:**
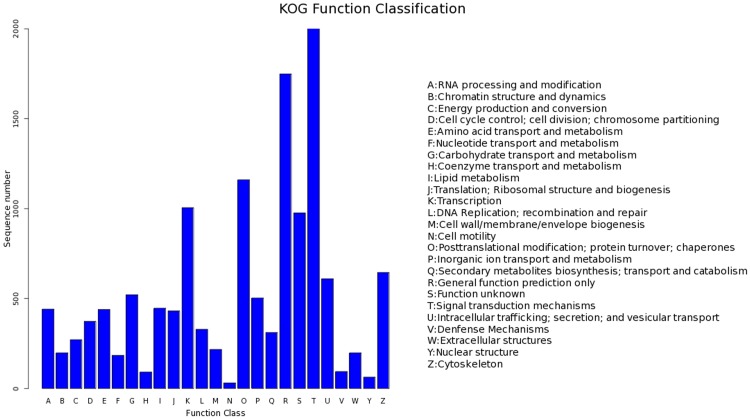
EuKaryotic Orthologous Groups (KOG) function classifications of *Patinopectin yessoensis*.

KOG analysis was carried out to provide a deeper understanding of the functions of the unigenes. About 13,905 unigenes were classified into 25 functional categories. The category of ‘signal transduction mechanisms’ contained the largest number of unigenes (2,619, 18.83%) (Fig. 1), followed by the ‘general function prediction only’ cluster (1,749, 12.58%) and the ‘posttranslational modification; protein’ cluster (1160, 8.34%).

The categories of greatest interest in the present study were inorganic ion transport and metabolism (503, 3.61%), defense mechanisms (94, 0.68%) and signal transduction mechanisms (2,619, 18.83%). Because the genes in these categories were likely related to immune function, biomineralization, and shell coloring, these categories should be considered for the development of molecular markers in the *P*. *yessoensis* breeding programs.

### Gene Ontology (GO) classification

**Fig 2:**
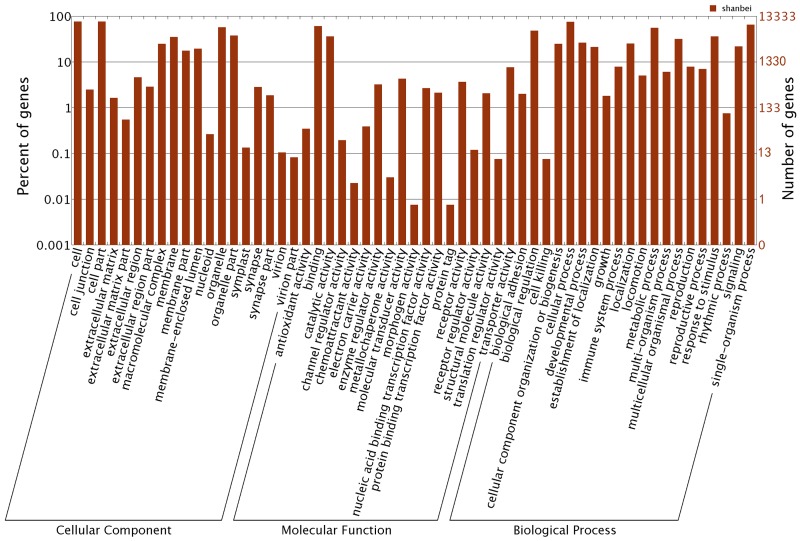
Gene Ontology classifications of assembled sequences in *Patinopectin yessoensis* transcriptome.

In addition to KOG analysis, we conducted GO analysis on the basis of sequence similarities to known proteins in the Nr databases. This analysis provided hierarchical relationships that represent information on molecular functions, cellular components, and biological processes. A total of 13,333 unigenes were annotated by GO analysis with one or more GO term (Fig. 2), for a total of11,037 GO assignments. Among these, 10,457 unigenes were annotated to the ontology of molecular functions, 11,963 to biological processes, and 10,997 to cellular components. For cellular components, the major represented categories were cell (GO: 0005623) and cell part (GO:0044464). For biological processes, cellular processes (GO: 0009987) was the most represented GO term, followed by single-organism processes (GO: 0044699).Genes involved in other important biological processes, such as growth, immune system processes, and biological regulation were also identified. Furthermore, we also found a number of unigenes that were involved in interesting categories, such as biomineralization and pigmentation, which may play a role in the shell and shell color formation (S3 Table). Regarding molecular functions, genes involved in binding (GO: 0005488) and metabolic process (GO: 0008152) were highly represented.

### Kyoto Encyclopedia of Genes and Genomes (KEGG) classification

**Fig 3:**
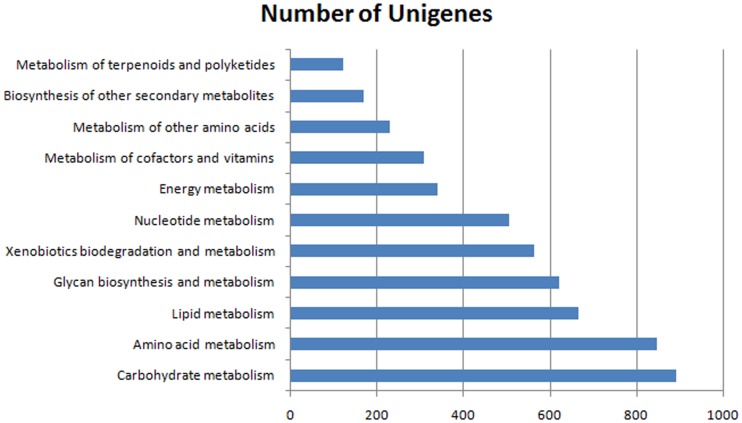
Categories classified by Kyoto Encyclopedia of Genes and Genomes (KEGG).

KEGG pathway analysis was carried out on the assembled unigenes to reveal the biochemical pathways operating in *P*. *yessoensis*. The results annotated 8,500 unigenes into 292 different pathways (Table 3). Among these, metabolic pathways contained the largest number of unigenes, and there were three major subgroups involved in carbohydrate metabolism, amino acid metabolism, and lipid metabolism, respectively (Fig. 3). Another pathway of interest included the subgroups of betalain biosynthesis, flavonoid biosynthesis, indole alkaloid biosynthesis, anthocyanin biosynthesis, calcium signaling, and carotenoid biosynthesis. These pathways are all related to the synthesis of biological pigments, so we inferred that the pathways probably play significant roles in biomineralization and shell coloring.

**Table 3:** Kyoto Encyclopedia of Genes and Genomes (KEGG) pathway mapping for Patinopectin yessoensis.

KEGG Pathways	Sub-Pathways	Number of Unigenes
**Metabolism**	Carbohydrate metabolism	893
	Energy metabolism	341
	Lipid metabolism	667
	Amino acid metabolism	848
	Metabolism of other amino acids	231
	Glycan biosynthesis and metabolism	622
	Metabolism of cofactors and vitamins	311
	Metabolism of terpenoids and polyketides	123
	Biosynthesis of other secondary metabolites	171
	Xenobiotics biodegradation and metabolism	564
	Nucleotide metabolism	506
**Genetic Information Processing**	Transcription	406
	Translation	608
	Folding, sorting, and degradation	909
	Replication and repair	279
**Environmental Information Processing**	Membrane transport	84
	Signal transduction	1,665
	Signaling molecules and interaction	769
**Cellular Processes**	Transport and catabolism	971
	Cell motility	253
	Cell growth and death	944
	Cell communication	945
**Organismal Systems**	Immune system	1,332
	Endocrine system	891
	Circulatory system	277
	Digestive system	851
	Excretory system	257
	Nervous system	623
	Sensory system	332
	Development	350
	Environmental adaptation	192

### Functional genes involved in growth, immunity, and biomineralization

The important economic traits of growth and immunity have been the focus of much work on economically valuable marine shellfish[19–21]. The unigene and annotation information from Nr, Swiss-Prot, GO, and KEGG all provide valuable genomic resources for *P*. *yessoensis* and will enable further study of the molecular basis of growth, immune function, and shell biomineralization.

Many environmental factors, including water temperature and salinity, hormone effects, and molecular factors, affect the growth and development of *P*. *yessoensis*[22–23]. In the present study, we identified a series of genes related to growth, such as transforming growth factor (TGF)-beta regulator 1, TGF-beta receptor-associated protein 1, vascular endothelial growth factor and receptor, acidic fibroblast growth factor intracellular-binding protein, bone morphogenetic protein, connective tissue growth factors, epidermal growth factor and receptors, fibroblast growth factor and receptors, and mitogen-activated protein kinase.

The living environment of *P*. *yessoensis* is filled with various kinds of parasites and pathogens, but scallops have a fairly complete immune system that protects them from infections and other damage[24]. Unlike vertebrates, shellfish have no specific immune lymphoid cells or antibodies, and their cellular immunity relies on phagocytosis[25]. We classified genes involved with immune function, including those that produce lysozyme, acid phosphatase, alkaline phosphatase, beta-glucuronidase, lectin, superoxide dismutase, catalase, toll-like receptor, heat shock protein, stress-associated endoplasmic reticulum protein, and stress-activated protein kinase JNK.

Among these genes, the ones that produce lysozyme include the lysosomal enzymes acid phosphatase, alkaline phosphatase, and β-glucuronidase. During phagocytosis, lysosomal enzyme is released into the blood serum [26]. Lysozyme is a ubiquitous antibacterial enzyme that lyses bacterial cell walls and occurs in many tissues and secretions. Extensive studies have suggested that it plays an important role in the body’s defenses against infection [27]. Acid phosphatase and alkaline phosphatase have direct antibacterial activities and could function as regulatory factors to influence the phagocytic process. Lectins have been reported in many marine bivalves, such as clams, oysters, and mussels. They are important pattern recognition receptors that recognize pathogen-associated molecular patterns and deliver appropriate signals to the cell to alert the innate immune system [28].

For KEGG analysis, a total of 1,332 sequences were classified into 15 immune-response pathways, including the chemokine signaling pathway, complement and coagulation cascades, antigen processing and presentation, the toll-like receptor signaling pathway, the NOD-like receptor signaling pathway, the RIG-I-like receptor signaling pathway, Fc gamma R-mediated phagocytosis, the cytosolic DNA-sensing pathway, and leukocyte transendothelial migration. These pathways may play important roles in the body immunity of *P*. *yessoensis*.

Shells, one of the most interesting features of bivalves, are secreted by the mantle in a process called biomineralization. Chitin is an important component in bivalve nacre formation that promotes the biomineralization process by forming the framework for other macromolecular components [29]. We identified four genes related to chitin biosynthesis: chitin synthase, chitin deacetylase isoform A, chitinase A1, and chitinase domain-containing protein 1. Calcium metabolism also plays an interesting role in shell formation, as more than 95% of the nacre’s weight is calcium carbonate [30]. We identified several genes related to calcium metabolism, such as calreticulin, calmodulin, calmodulin-binding protein, calcitonin receptor, calcium-binding protein, calcium and integrin-binding protein, and calcyclin-binding protein.

Functional analysis using the Illumina sequencing database identified genes that are potentially related to growth, immunity, and biomineralization. Further experiments should demonstrate the functions and expression patterns of these candidate genes and should analyze their potential roles in growth and immune function of *P*. *yessoensis* of different shell color lines.

### Differential gene expression among the four shell color strains

A total of 21,859 annotated unigenes which did not match prokaryotic and protozoan sequences were used as the reference transcriptome for mapping each set of reads from the different shell morphs. The reads of the four *P*. *yessoensis* transcriptome libraries generated by Illumina paired-end sequencing were mapped to assembly reference sequences. The mapping rates exceeded 93% for total reads mapped and 86% for total unique reads mapped (Table 4).

**Table 4:** Statistics regarding alignment of sequences.

	P1	P2	P3	P4
Number of reads	41,865,770	30,897,906	57,955,052	35,802,648
Number of reads mapped	39,189,962	28,733,671	54,285,922	33,500,633
% of reads mapped	93.61%	93.00%	93.67%	93.57%
Total number of unique reads	36,699,002	26,871,380	50,633,697	31,232,858
Total % of unique reads	87.66%	86.97%	87.37%	87.49%

### Variation of gene expression among different shell color lines of *P*. *yessoensis*

**Fig 4:**
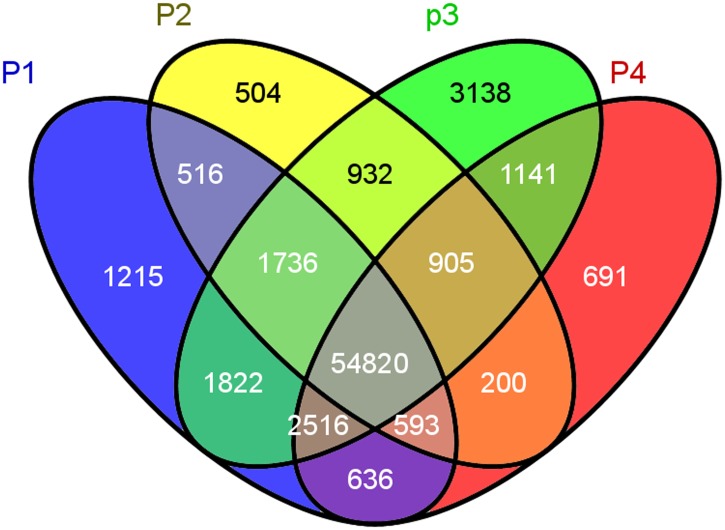
Venn diagram of the expressed genes along the four different *Patinopectin yessoensis* transcriptomes. A total of 54,820 genes were co-expressed among the four samples. The Venn diagram serves as an interactive tool for comparing the list, which was plotted using Venn.

**Fig 5:**
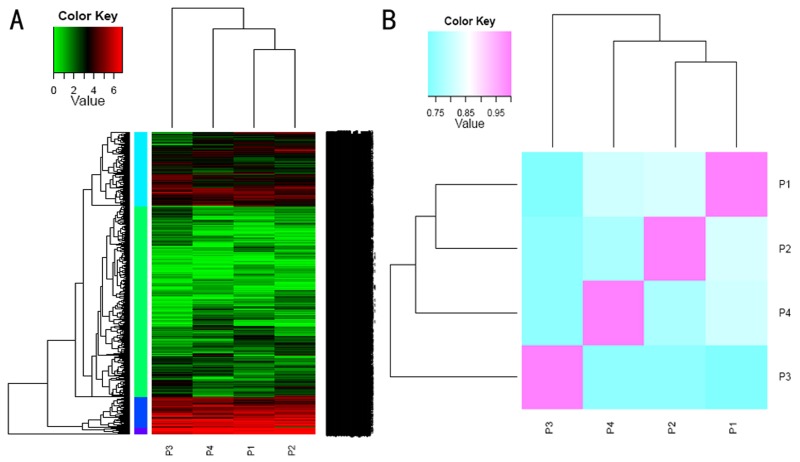
Comparisons of transcriptional profiles across samples. (a) Hierarchical clustering of transcripts and samples. (b) The hierarchically clustered Spearman correlation matrix resulting from comparing the transcript expression values for each pair of samples.

To characterize the transcriptome of *P*. *yessoensis* strains with different shell colors, we collected samples from ordinary strains of *P*. *yessoensis* (left and right shell mantle), the ‘Ivory’ strain, and the ‘Maple’ strain (left shell mantle). These four samples represented three distinct strains of *P*. *yessoensis*, and we compared the gene expression of these four transcriptomes. A total of 63,854, 60,206, 67,010, and 61,502 genes were detected in the P1, P2, P3, and P4 libraries, respectively, and 54,820 genes were co-expressed among all four transcriptome libraries (Fig. 4). Of these, 3,138 genes were exclusively expressed in P3, which is more than in any other library, and 1,215, 504, and 691 genes were detected only in the P1, P2, and P4 transcriptomes, respectively (Fig. 4). Transcriptomes of P1 and P2, which come from the “original” strain of *P*. *yessoensis*, exhibited high similarity and were grouped first (Pearson’s correlation r = 0.843); next, P4 joined in them as the first group, and finally, P3 formed the second group (Fig. 5). The gene expression profiles among the mantle of the P2 library, which produced a white shell (the right shell mantle of the ordinary strain) were similar to those of P3 (shell mantle of the ‘Ivory’ strain) (Pearson’s correlation r = 0.761), as expected. The variation observed in the transcriptomes of different *P*. *yessoensis* strains reflects the overall effects of all non-genetic and genetic factors, and it is clear that more changes in gene expression occur in the ‘Ivory’ strain of *P*. *yessoensis*.

**Fig 6:**
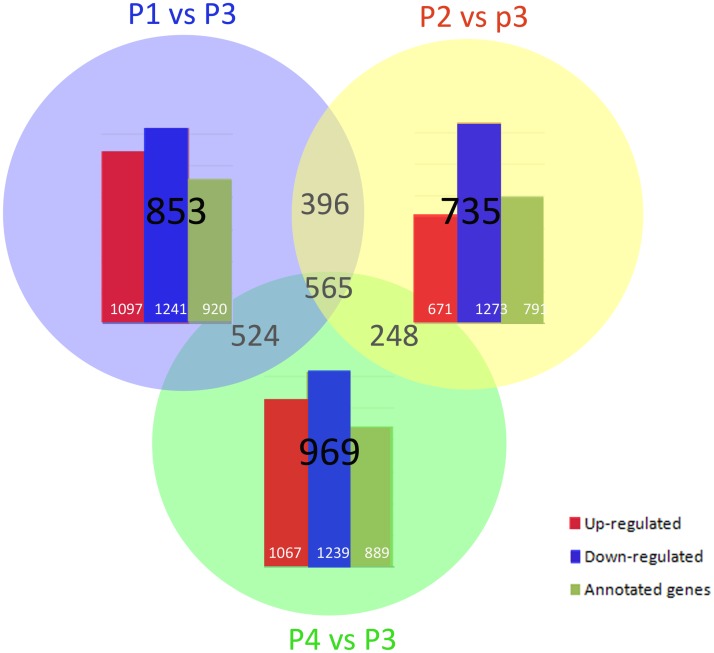
Changes in gene expression profiling among the different treatment. Up-regulated (red), down- regulated (blue), and annotation (green) unigenes were quantified and presented by histogram. Comparisons of DEG in P1/P3, P2/P3, and P4/P3 are presented by Venn chart.

**Fig 7:**
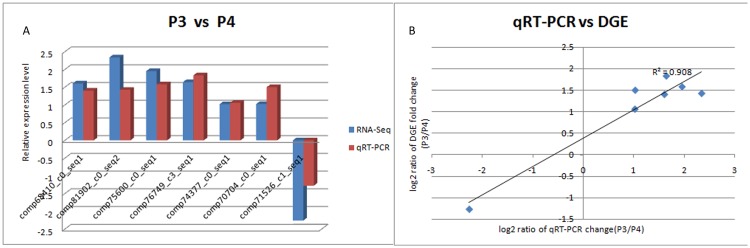
Quantitative reverse-transcription-PCR validation of differentially expressed gene (DEG) results. (a) Fold changes measured by mRNA-Seq and real-time RT-PCR. (b) Correlation between the expression fold change level of DEG between P3 and P4.

In order to determine the differences in *P*. *yessoensis* from different shell color lines more accurately, we used DEGseq [31] to screen the differentially expressed genes (DEG) (*p*< 0.05) with a relation model that chose P3 as the control group and compared it with the other three transcriptome libraries. We found 2,338 differentially expressed genes with 1,097 up-regulated genes and 1,241 down-regulated genes in the P1 library, compared with the control group library (P3). A total of 1,944 and 2,306 DEGs were also identified in the P2 and P4 libraries, respectively, compared with P3 (Fig. 6). To confirm whether our sequencing and analysis were reliable and valid, we randomly selected seven DEGs and measured their expression in the same RNA sample of P3 and P4 by real-time reverse transcription (RT)-PCR. All seven genes showed uniformly consistent results in RT-PCR and transcriptome sequencing (Fig. 7), which indicates that transcriptome sequencing was reliable and we can make reasonable deductions from the functional enrichment analysis of the DEGs.

### Functional analysis of differentially expressed genes

In a comparison of the DEGs with a reference gene database (21,859 annotated unigenes), 920, 791 and 889 DEGs were annotated, in P1/P3, P2/P3, and P4/P3, respectively (Fig. 6). However, a large number of DEGs could not be annotated, including some highly expressed genes. In order to better understand the functions of DEGs, GO and KEGG pathway enrichment analysis was carried out on the DEGs.

Betalain biosynthesis (ko00965, *p* < 0.01) and tyrosine metabolism (ko00350, *p* < 0.01) were significantly enriched in the DEGs of P1/P3, P2/P3, and P4/P3; Moreover, across all three comparisons (P1/P3, P2/P3, and P4/P3) 565 DEGs (248 annotated genes) were consistently differentially expressed. As betalain biosynthesis and tyrosine metabolism were also significantly enriched among these genes, we hypothesize these two pathways were involved in coloration. Betalains are natural pigments, nitrogen-containing water-soluble compounds derived from tyrosine, that confer yellow/red colors [32]. The intermediate products derived from the oxidation of tyrosine and indoles constitute melanin, which is an irregular light-absorbing polymer [33]. Furthermore, we found that tyrosinase-like protein existed in both the betalain biosynthesis and tyrosine metabolism pathways, and this gene was expressed to a greater extent in the mantles with red shells. Whether tyrosinase-like protein is related to the formation of red shells is an interesting question that will be explored in future study.

The shell is formed by the mantle in a process that is similar to pearl formation [34]. Although there are different views on the relationship between the amount of trace metals and the color of the shell and pearl, researchers generally believe that trace metals are linked closely with shell and pearl coloring [34]. Some amount of the trace metals is stored in the aragonite in its ionic state, and the other trace metals unite with porphyrins to form metal-porphyrins, resulting in different colors in shells and pearls [35–36]. In our study, the pathways of porphyrin and chlorophyll metabolism (ko00860) were also enriched in the DEGs from the P3 library over those in the P1, P2, and P4 libraries. Some ferritin, which has a lower expression in the mantle of ‘Ivory’ strains of *P*. *yessoensis*, was also found in these pathways.

In order to determine which metal ions mainly affected the color of the shell in *P*. *yessoensis*, we compared the amount of metal elements in red shells and white shells. The amounts of Fe and Zn in red shells were significantly higher, 3.04 and 2.41 times these amounts of white shells, respectively (Table 5). Through analysis of these DEGs, we obtained several genes that are involved in metal transport. It has been shown that ferritin controls the concentration and distribution of iron in mollusk shells, sculpting shell morphology and coloration [37–38]. Metalloreductase STEAP2, divalent metal transporter 1, and zinc transporter ZIP12 also play important roles in Fe^2+^ and Zn^2+^ transport [39–42]. In addition, these genes in the P1 library (representing the reddish-brown shell) existed at higher levels than in the other libraries (representing the white and red-and-white shells). The measured results of trace elements were confirmed by the transcriptome analysis result, and both of these analyses suggest that formation of the reddish-brown shell requires more iron and zinc ions. Moreover, iron-porphyrins and zinc-porphyrins also produce red and pink colors, respectively [43].

**Table 5:** Amounts of trace elements measured in Patinopectin yessoensis (mg/kg).

Sample	Mn	Cu	Zn	Fe	Mg
Left shell of ‘Ivory’ strain	17.10	> 0.10	4.02	18.40	938.00
Left shell of ordinary strain	19.20	> 0.10	9.70	55.6	964.00

The DEG identification can be improved by searching for SNPs that are associated with these genes. Further identification of SNPs within these genes could explain shell color variation between different shell color lines and could provide a foundation for further studies into the molecular mechanisms controlling mollusk shell coloration.

### Detection of SSRs and SNPs

Transcriptome is a valuable resource for the effective and convenient development of genetic markers. Both SSRs and SNPs are valuable molecular markers for the *P*. *yessoensis* breeding program and offer the greatest potential for identifying functional genes of economically important traits. Approximately 3 to 7% of expressed genes contain putative SSR motifs [44], and SSR markers have been widely used to construct genetic maps in marine animals[45]. In our study, we performed a general screen for SSRs on the sequences that were integrated from the four *P*. *yessoensis* transcriptomes. The screening produced a total of 5,772 SSRs with 142 motifs. Dinucleotides (2,639) and trinucleotides (2,989) were major types of SSRs, followed by tetranucleotides (131) and pentanucleotides (13). The most common SSR type, considering sequence complementarity, was AAC/GTT (1,191), followed by AG/CT (1,090), AT/AT (901), AAG/CTT (506), AAT/ATT (317), and ATC/ATG (274).

**Fig 8:**
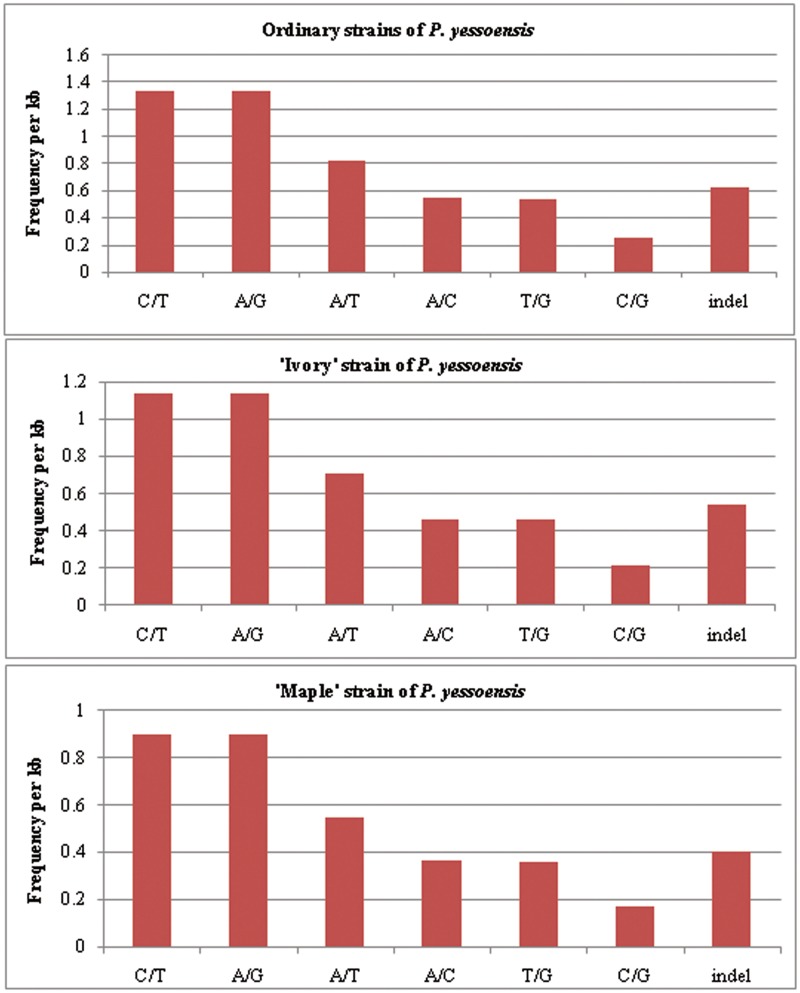
Frequency distribution of single nucleotide polymorphisms identified in *Patinopectin yessoensis* transcriptomes from different shell color lines.

The ESTs generated in our study through Illumina sequencing originated in three different strains of *P*. *yessoensis* samples. We screened for SNPs that were present in all three strains. In ordinary strains of *P*. *yessoensis*, a total of 464,197 high-quality SNPs were generated, among which 228,136 were transitions, 183,247 were transversions, and 52,814 were in indels. In ‘Ivory,’ there were 395,646 high-quality SNPs, among which 192,782 were transitions, 156,693 were transversions, and 46,171 were indels. In ‘Maple,’ there were 310,649 high-quality SNPs, among which 153,280 were transitions, 122,883 were transversions, and 34,486 were indels (Fig. 8).

The SSRs and SNPs generated from this study constitute an important and valuable resource for further studies on the analysis of marker development, genetic linkage mapping, and interesting traits in *P*. *yessoensis*.

## Conclusions

We performed *de novo* transcriptome sequencing for *P*. *yessoensis* from different shell color lines using the Illumina platform. A large number of candidate genes potentially involved in growth, immunity, and shell biomineralization were identified and are worthy of further investigation. In addition, genes that are differentially expressed in different shell color lines of *P*. *yessoensis* were identified and functionally annotated using the GO and KEGG databases. This information provides potential molecular targets in shell coloring and metal transcription. A large number of SNPs and SSRs identified in this study will provide a valuable material foundation for future genetic linkage analysis and will help to further aquaculture breeding programs for this species.

## Materials and Methods

### Ethics statement

Not applicable. Our research did not involve human participants or samples. Housing and care of *P*. *yessoensis* and collection of tissue samples for use in the experiments described were conducted in accordance with the International Guiding Principles for Biomedical Research Involving Animals (http://www.cioms.ch/frame 1985 texts of guidelines.html).

### Sampling of and RNA extraction from mantle tissues of *P*. *yessoensis*

In order to obtain more and better information from the transcriptomes of different color lines of *P*. *yessoensis* by Illumina paired-end sequencing, we collected 2-year-old live individuals of ordinary, ‘Ivory,’ and ‘Maple’ strains of *P*. *yessoensis*. We maintained all of these scallops in the same cultivation environment, at the Key Laboratory of Mariculture, Ministry of Agriculture, Dalian, Liaoning Province, China, in 2013. The scallops were healthy and homogeneous in size, with shell weights of 12.64 ± 0.32 g and shell lengths of 4.82 ± 0.23 cm. The mantles of the left and right shells were randomly collected from at least three independent ordinary strains of *P*. *yessoensis* as samples (Sample P1 and Sample P2) and were used for RNA extraction, as were the mantles of the left shell of ‘Ivory’ and ‘Maple’ (Sample P3 and Sample P4). Total RNA was isolated from each sample using an RNAprep pure tissue kit (TIANGEN, CHN), following the manufacturer’s protocol. The quantity and quality of total RNA were confirmed using the NanoDrop2000 spectrophotometer (Thermo Scientific, Wilmington, DE, USA) and gel electrophoresis.

### cDNA library preparation for Illumina sequencing

Each paired-end cDNA library was generated using the Genomic Sample Prep Kit (Illumina, San Diego, CA, USA) according to the manufacturer’s instructions. The quality of the library was assessed using Agilent 2100 Bioanalyzer (Agilent Technologies, Santa Clara, CA, USA), and cluster amplification was performed using the TruSeq PE Cluster Kit and a cBot (Illumina). The four cDNA libraries were sequenced with a paired-end module at Beijing Institute of Genomics, Chinese Academy of Sciences, Beijing, China.

### *De novo* assembly of Illumina sequencing reads

Before assembly, the 101-bp raw paired-end reads were filtered to obtain high-quality clean reads by removing adaptors, PCR duplicates, and low-quality sequences (reads with a base quality below 20). Due to the lack of genetic information about *P*. *yessoensis*, the clean reads from four libraries were mixed together as a reference database, and *de novo* assembly of these clean reads was performed using the short reads assembling program Trinity (default parameters were used) [15]. All assembled sequences were compared with the NCBI non-redundant protein database, Swiss-Prot database, the KEGG pathway, and the KOG database using BlastX with an E-value of less than 10^–5^, and the best aligned results were used to decide the sequence direction of unigenes.If the results of different databases conflicted, a priority order of Nr, Swiss-Port, KEGG, and KOG was followed. When a unigene did not align to any of the databases, ESTScan was used to predict the sequence direction [46].

### Gene annotation and classification

Functional annotations were performed by sequence comparison with public databases. The unigenes were compared against the Nr, Swiss-Prot, KEGG, and KOG databases by BlastX (E-value < 10^–5^), and the highest sequence similarity with the given unigenes was defined as the unigene functional annotation and used in the following analysis. ForNr annotation, we used the Blast2GO program to obtain the GO annotations of the unigenes (E-value < 10^–5^) [47]. We also used WEGO software to conduct GO functional classification for all unigenes [48]. Unigenes were aligned to the KOG database to predict and classify possible functions, and pathway annotation was performed using Blastall software against the KEGG database [49]. In addition, any elements of the unigenes that matched to prokaryote and protozoan databases were considered contaminants and were removed from the analysis.

### Comparison of the transcriptomes of four cDNA libraries

The clean reads from four cDNA libraries were mapped on the reference database by BWA software with the default parameter value [15], and the mapping rates were all around 80%. The RPKM (reads per kilobase per million reads) [50] was applied to measure the gene expression levels. We used the calculated gene expression to compare the differences in gene expression between four samples. We performed pair-wise comparisons of the gene expression of these four transcriptomes using the DEGseq [31] package, and *p* < 0.05 was used as the threshold to screen the differentially expressed genes. Then, the hierarchical clustering of transcripts and samples was used to show the relative expression levels of each transcript in each sample, and a comparison of the transcript expression values for each pair of samples was used to show the hierarchically clustered Spearman correlation matrix [51]. For pathway and GO enrichment analysis, we selected *p* < 0.05 as a threshold to identify the significantly enriched KEGG and GO terms.

To assess the reliability of our sequencing and analysis by real-time RT-PCR, we used the same RNA samples for sequencing from P3 and P4, and we synthesized cDNA using the PrimeScript RT reagent Kit (TaKaRa, DL, CHN). Real-time PCR was performed in triplicate according to the manufacturer’s instructions by using the SYBR Premix Ex Taq (Tli RNaseH Plus) Kit (TaKaRa, DL, CHN) on an ABI 7500 real-time PCR system and analyzed by the 2^-ΔΔCt^ method using β-actin as the reference control [52]. All the primer sequences used for RT-PCR are listed in S4 Table.

### Trace metal composition in the reddish-brown shell and white shell

Samples of red and white shell were collected from the left shells of live individuals of ordinary and ‘Ivory’ strains of *P*. *yessoensis* from the Key Laboratory of Mariculture, Ministry of Agriculture, Dalian, Liaoning Province, China, in 2013. The sampled *P*. *yessoensis* individuals were healthy and homogeneous in size, with a shell weight of 4.64 ± 0.32 g and a shell length of 2.82 ± 0.23 cm. We opened the scallops with a scalpel, ensured the integrity of the shell, and washed the sample with deionized water. Then, these shells were oven-dried at 65°C for more than 24 h, the reddish-brown shell and the white shell were transferred to separate mortars, and both were ground to a fine powder. Finally, the amounts of the trace metals Mn, Cu, Zn, Fe, and Mg from both samples were evaluated using atomic absorption spectrometry.

### The identification of SNPs and SSRs

All types of SSRs were identified and localized microsatellite motifs were identified using MIcroSAtellite (MISA, http://pgrc.ipk-gatersleben.de/misa/) software. The criteria were that sequences had at least six repeats of dinucleotide and five repeats of all other motifs (from trinucleotide to hexanucleotide).

As the four transcriptomes of differently colored shell lines belong to three strains of *P*. *yessoensis*, we screened for the presence of SNPs in all three strains. We identified potential SNPs using the GATK program [53] and filtered the results using two parameters (SNP quality > = 30 and reads depth > = 5), producing 467,098 putative SNPs.

## Supporting Information

S1 Table Summary statistics of *Patinopectin yessoensis* sequencing raw reads. (XLSX)

S2 Table Sequences with significant BLAST matches against biological functions. (XLSX)

S3 Table Statistics of the Gene Ontology (GO) term involved in immune, biomineralization, and pigmentation. (XLSX)

S4 Table Statistics on primer sequences. (XLSX)
